# Measurement of virulence in *Zymoseptoria tritici* through low inoculum-density assays

**DOI:** 10.1016/j.fgb.2015.03.020

**Published:** 2015-06

**Authors:** Helen N. Fones, Gero Steinberg, Sarah Jane Gurr

**Affiliations:** Biosciences, College of Life and Environmental Sciences, University of Exeter, Stocker Road, Exeter EX4 4QD, UK

**Keywords:** *Zymoseptoria*, Lesion formation, Pycnidiation, Pathogenicity, Virulence

## Abstract

Hitherto, pathogenicity assays with mutants or wildtype variants of *Zymoseptoria tritici* have been based on pycnidial counts, following inoculation of host leaves with high density inoculum. Here, we present data which suggest that high inoculum densities may mask deficiencies in virulence due to symptom saturation. We describe a low inoculum-density method which obviates this problem. This method can also be used to (i) interrogate the process of lesion formation in *Z. tritici* (ii) determine whether individuals of the same or different genotypes co-operate or compete during the establishment of apoplastic infections (iii) dissect the determinants of virulence, by assessing a given strain’s stomatal penetration efficiency (SPE), its ability to spread within the apoplast and its pycnidiation efficiency. Such methodology can thus be used to investigate the reasons underpinning attenuated virulence in mutant or avirulent wildtype strains.

## 1 Introduction

During the advanced stages of *Zymoseptoria tritici* infection, wheat leaf tissues becomes chlorotic and necrotic lesions develop (Palmer and Skinner, 2002; Orton et al., 2011). These lesions display a characteristic diamond shape “dotted” with pycnidia. *Z. tritici* spreads throughout the apoplastic spaces of the epidermal and mesophyll layers of infected leaves, forming “basket-like” structures in the substomatal spaces. These develop into pycnidia (Kema et al., 1996; Howard & Duncan, 2000), which, on maturity, extrude asexual spores. The susceptibility of a particular wheat cultivar to a given *Zymoseptoria* strain can be determined by consideration of the degree of lesion coverage on the leaf (Rosielle, 1972), pycnidial density (Eyal and Brown, 1976) and disease height (Eyal et al., 1987).

Typically, wind-borne ascospores provide the initial fungal inoculum (reviewed in Suffert et al., 2011). Subsequent disease amplification throughout the wheat leaf canopy results from rainsplash distribution of pycnidiospores (reviewed in Suffert et al., 2011). Thus, co-infection of wheat plants is usual, with this epidemiological pattern of infection enabling a single wheat field to host up to 70 clonal strains (Shaw and Royle, 1989; Cohen and Eyal, 1993; Chen and McDonald, 1996; McDonald et al., 1999).

The population genetics of *Z. tritici* has been studied at different spatial levels, from field populations to within individual leaf disease lesions (Linde et al., 2002). Interestingly, there is evidence that greater genetic variation exists between lesions than within a given lesion (Linde et al., 2002). Indeed, 36% of lesions were found to carry only one fungal genotype, whilst many of the 64% that contained several genotypes were dominated by one particular strain. Given that lesions can coalesce or overlap on a susceptible wheat leaf (Rosielle, 1972; Linde et al., 2002), and noting the variation in the *Z. tritici* population at the field level (Shaw and Royle, 1989), this dominance of single genotypes within lesions is surprising. It suggests that many, if not most lesions, are initially formed as a result of a single stomatal penetration event followed by fungal spread within the apoplast.

Here, we test this ‘one penetration, one lesion’ idea by the use of a dilution series of fungal cell suspensions and a known inoculum volume. By comparing the response of lesion number, lesion size and the density of pycnidia within lesions to increasingly dense inocula, we determine (i) how many fungal individuals are needed to form a necrotic lesion and (ii) whether individuals co-operate inside the leaf, either to form bigger lesions or to fill more of the available sub-stomatal spaces with pycnidia.

Currently, pathogenicity assays with *Z. tritici* rely mainly on pycnidial counts. The method we describe suggests that deficiencies in virulence may be masked by the use of high inoculum densities. Moreover, this low inoculum density method enables us to separate out the events of fungal entry, host colonisation and pycnidial formation. It thus provides a way to interrogate and compare the process of infection between wild type and mutant strains.

## 2 Results & discussion: IPO323-Galaxie pathosystem

**Fig. 1:**
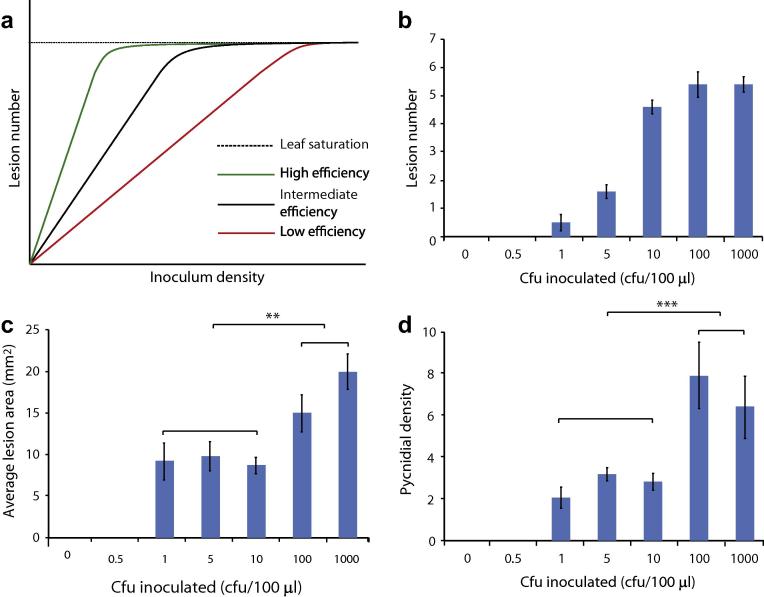
Lesion forming behaviour of IPO323 on Galaxie. (a) Expected response of lesion number to inoculum density: in the absence of other factors, lesions number would increase linearly according to the number of spores added, until a point is reached where the available leaf surface area is saturated and no further increase is possible. The slope of the linear response will depend upon the efficiency with which spores infect. (b) Actual response of *Zymoseptoria tritici strain* IPO323 lesion number on wheat cultivar Galaxie leaves to inoculum density. The expected linear response is seen at low inoculum densities, with saturation occurring between 100–1000 cfu/ml inoculum (*i.e.* 10–100 cfu per leaf). (c) Lesion area does not change with inoculum density until lesions begin to coalesce. (d) Pycnidial density is unaffected by changes in inoculum density until lesions begin to coalesce, when the number of pycnidia per mm^2^ of lesion area increases. 2 independent experiments. Values are means and error bars represent SE. *n* ⩾ 4 biological replicates. Asterisks indicate significant differences (*t-*tests) between marked groups at *α* = 0.05 (∗), 0.01 (∗∗) or 0.001 (∗∗∗). No stars indicates a non-significant result.

Galaxie wheat plants were inoculated with a dilution series of an IPO323 spore suspension as described (see Section 4). Lesions appearing on infected leaves were then enumerated. The use of a dilution series of fungal cell suspensions and a known inoculum volume, allows us to calculate the ratio of successful stomatal penetration events to number of cells applied. This can be described as stomatal penetration efficiency (SPE). If each inoculated cell, for example, breaches the leaf surface to produce one lesion, the application of 100 μl of a 50 cfu/ml cell suspension would be expected to produce 5 lesions on the inoculated leaf, whilst a 100 cfu/ml cell suspension would produce 10 lesions. However, with an SPE of 10%, this number would be reduced to an average of 0.5 or 1 lesion/inoculated leaf for 50 or 100 cfu/ml cell suspensions, respectively (Fig. 1a). Importantly, however, a linear relationship between inoculum density and lesion number would be expected only in the case that inoculated cells act independently to produce lesions. If multiple cells co-operate, either to enter the plant or to produce a macroscopic lesion, a different response curve would be seen.

**Fig. 2:**
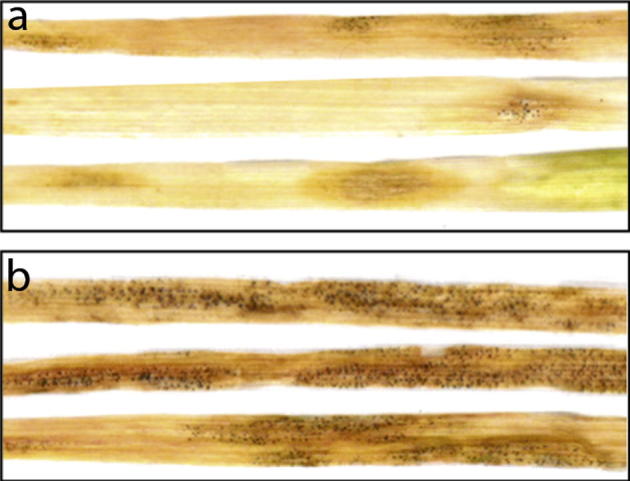
Lesions caused by *Zymoseptoria tritici* IPO323 on wheat cultivar Galaxie. Characteristic diamond shaped, pycnidia-bearing lesions on a lightly infected leaf (a), where position and extent of lesions remains clear despite necrotic collapse of leaf. (b) Coalescing lesions on heavily infected leaves (21 dpi).

To test this, we inoculated wheat cultivar Galaxie with *Z. tritici* strain IPO323. We found a linear response when the leaves were inoculated with 1–10 cells (100 μl of a 10–100 cfu/ml cell suspension; Fig. 1b). Over this range, this gives an SPE of around 50%, meaning that inoculation with 10 cells gives on average 5 lesions. Thus, lesions must be formed by a maximum of two inoculated cells. However, since an inoculum containing 1 cell per leaf (100 μl at density 10 cfu/ml) gives on average 0.5 lesions per leaf, it is more likely that one cell produces 1 lesion, with a 50% ‘success rate’ (SPE). At inoculum densities higher than 100 cfu/ml the surface of the 10 day old leaves used in this work became saturated and lesions began to meet and therefore to coalesce (Fig. 2). After this point, individual lesions cannot be distinguished accurately. As a result, the increase in lesion number slows, then ceases, and lesions begin to appear to be larger (Fig. 1c) as a result of summation of their areas. In the IPO323-Galaxie pathosystem, this occurs when lesion number reaches between 5 and 50 per leaf (ie when 10–100 cfu are inoculated onto each leaf). Interestingly, after this coalescence threshold, lesions contain more pycnidia per mm^2^ (Fig. 1d). This indicates that when individuals meet within the apoplast, the result is more efficient filling of the sub-stomatal apertures with pycnidia.

The degree of infection is usually determined as pycnidial counts per mm^2^ of leaf and, generally, experiments are performed using high inoculum cell densities, typically around 10^5^ cfu/ml. At such high inoculum densities, the leaf is covered by lesions, so that individual lesions coalesce and cannot be distinguished. Thus, even quite large differences in either fungal penetration ability (reflected in numbers of lesions, Fig 4a and b), or ability to spread within the apoplast (host colonisation; reflected in the size of individual lesions, Fig. 4a and d), may be less visible at high inoculum densities.

Here, we show that lesion number, size, and pycnidial density remain steady with inoculation of 100 or 1000 cells per leaf. Higher inoculum levels of 10^5^ cfu/ml, which here translate to adding 10,000 cells per leaf, well over the lesion coalescence threshold, may therefore mask changes in virulence of up to a thousand-fold. Furthermore, only those virulence attributes which are important for pycnidial production and which are independent of leaf entry or apoplastic spread (Fig. 4c), will be unaffected by this masking phenomenon. For this reason, we recommend the adoption of much lower inoculum density to assay pathogenicity and virulence in *Z. tritici.*

It is important to note that the results shown here demonstrate the potential of this method but cannot be considered universally applicable. An SPE of 50% is high and indicates that our growth and infection conditions are perhaps close to optimal for *Z. tritici.* A large number of factors, including fungal pre-culture times and growth conditions, use of different fungal strains and wheat varieties, as well as wheat growth conditions, will alter the precise SPE or fungal growth rate in the apoplast, and thus optimal inoculum density. Although our protocol is given in detail below, we therefore recommend that researchers obtain their own estimate of the maximum inoculum density that can be used in their conditions; that is conditions which give an outcome which falls below the coalescence threshold.

## 3 Conclusions

Previous studies have tended to use high inoculum densities to compare mutant and wildtype strains of *Z. tritici.* Here, we show that there is a risk, with this methodology, of overlooking significant differences in virulence. The method introduced here overcomes this difficulty. When a mutant is found to show a defect in virulence, this method can also be used to determine at which stage of the infection process the defect occurs. The three stages of infection are (1) entry into the host, (2) colonisation of the host and (3) formation of pycnidia (Steinberg 2015)

The methods described herein allow us to differentiate between these three failings at each of these stages relatively simply. Provided the inoculum density used is below the lesion coalescence threshold, then: A defect in host entry would show reduced numbers (or complete failure) of lesions and pycnidia (Fig. 4a and b). A defect in host colonisation will lead to smaller lesions than those formed by the wildtype (Fig. 4a and c). Finally, a defect in the formation or maturation of pycnidia will produce lesions with lowered pycnidial density or no pycnidia (Fig. 4a and d). Thus, this method could strengthen claims about the nature and importance of specific genes with respect to their role in the fungal life-cycle or in pathogenesis.

Finally, this method could be used to elucidate the interaction between multiple co-infecting strains, and thus is highly relevant for field-based studies. Co-inoculation may give rise to different values for SPE than observed for the strains individually. This would suggest differing degrees of co-operative infection between inoculated strains or, indeed, inhibition of germination by a dominant strain. An increase in the inoculum density needed to reach the lesion coalescence threshold would indicate inhibition of apoplastic colonisation by competing strains, as would observing a decrease in average lesion size. Finally, changes in pycnidial density within co-infected lesions would indicate competition or collaboration between strains in filling substomatal apertures or forming pycnidia. Such data could be of importance in understanding the dynamics of multi-strain infections, and thus of *Zymoseptoria* infections in the field (Chen and McDonald, 1996; Linde et al., 2002).

## 4 Methods

### 4.1 Plant inoculation methods

• Wheat seeds (*Triticum aestivum*, cultivar Galaxie, Fenaco, Bern, Switzerland) are sown on John Innes No.2 soil and maintained in growth chambers at 20 °C and 70% RH, with 10 h illumination per day for 10 days prior to infection.
• *Z. tritici* strain IP0323 (Kema and van Silfhout, 1997) is streaked onto YPD agar from stocks maintained at −80 °C, and incubated at 18 °C for 3 days to give heavy “yeast-like” growth on the agar surface.
• A small amount of this fungal material is transferred to 1 ml of 0.1% (v/v) Tween 20 solution using a pipette tip and vortexed briefly to suspend the cells evenly.
• This suspension is passed through two layers of sterile Miracloth (Calbiochem, Nottingham, *UK*).
• 10 μl of this suspension is then transferred to a haemocytometer and cfu/ml estimated.
• The filtered suspension is diluted to a cfu count of 5 × 10^6^/ml. From this, serial dilutions are made to give 10^5^, 10^4^, 10^3^, 100, 50, 10 and 5 cfu/ml suspensions.
• To inoculate the wheat plants, 50 μl of cell suspension is pipetted onto the base of a leaf (held horizontally) and spread evenly towards the leaf tip, using a gloved finger.
• The leaf is allowed to dry and then a further 50 μl of cell suspension is added in the same way
• Each fully expanded leaf of each wheat plant is inoculated, excepting the cotyledon, (usually 2 or 3 leaves for 10 day old cv. Galaxie plants).
• Inoculated leaves are marked with indelible pen at the base to allow the precise inoculated area to be defined, accounting for future leaf growth.
• Inoculated leaves are allowed to dry partially, preventing run-off of the cell suspension.
• Infected plants are bagged individually, to increase humidity levels, and to prevent cross-contamination and returned to the growth chamber.
• After 72 h, the bags are removed.
• Plants are maintained until 18 dpi.
• At least three independent plants are used per cell suspension for each repeat of the experiment.

### 4.2 Image collection and analysis

**Fig. 3:**
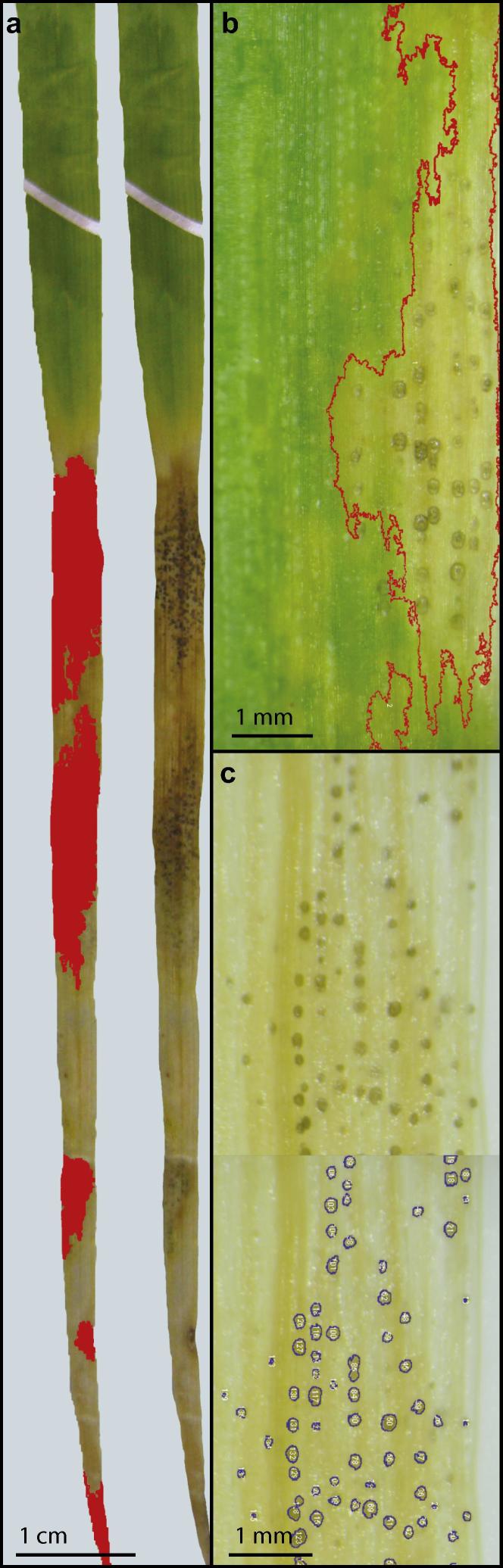
Threshold based selection of lesions and pycnidia using ImageJ software. (a) Infected leaf bearing five distinct lesions before (top) and after (bottom), thresholding based on hue and brightness in HSB colour space, allowing selection of lesions (b). This methodology can be applied to fully necrotic leaves (21 dpi; a) and to green or chlorotic leaves where necrosis is seen only at the lesions (18 dpi; b). After selection, lesion area can be measured. (c) Selection of pycnidia using thresholding, followed by particle analysis. The particle analysis tool allows criteria for circularity and size of pycnidia to be set, giving greater accuracy than thresholding alone, and provides a count of selected pycnidia.

**Fig. 4:**
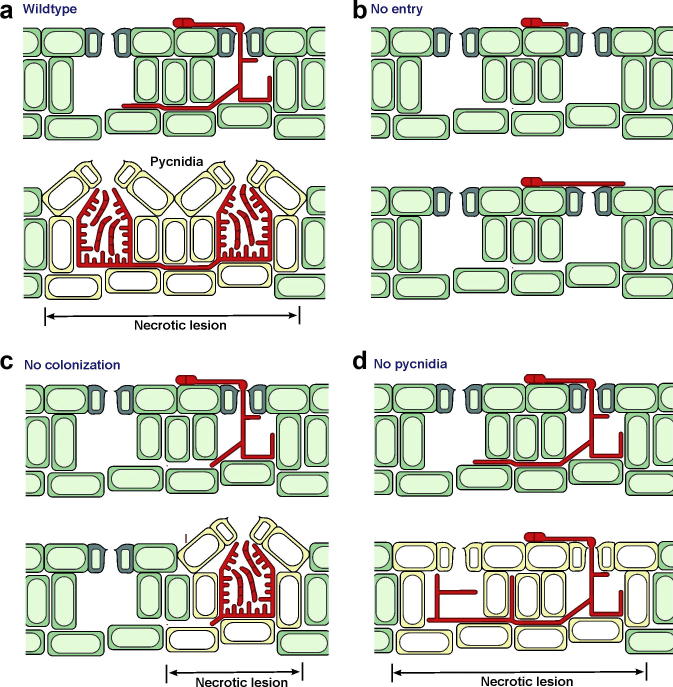
Symptom development in wildtype and putative mutant strains. (a) Schematic drawing of the early (upper panel) and late stages of infection (lower panel) of wildtype *Z. tritici*. Infection of wheat leaves can be seen as a 3 step process, starting with invasion of the plant *via* stomata, followed by colonization of the host apoplast and ending with the formation of pycnidia in necrotic lesions during the necrotrophic phase (Steinberg, 2015). (b) Schematic drawing of early stage of infection (upper panel) and late stage of infection (lower panel) of a mutant, defective in the entry into the host. Neither lesions nor pycnidia are formed. (c) Schematic drawing of early stage of infection (upper panel) and late stage of infection (lower panel) of a mutant, unable to colonise the plant apoplast. The fungus enters the stomatal cavity, but infection remains local as it is not able to spread within the leaf tissue. Local necrosis is expected to occur, forming small lesions. (d) Schematic drawing of early stage of infection (upper panel) and late stage of infection (lower panel) of a mutant, unable to form pycnidia. The fungus enters and hyphae colonize the mesophyll. Necrosis might occur, but no pycnidia are formed. Figure modified from Steinberg (2015).

• At 21 dpi, infected leaves are excised from the plants and photographed.
• These photos are used to analyse lesion size and number.
• Photographs are thresholded in HSB (Hue-Saturation-Brightness) colour space using ImageJ software (Abràmoff et al., 2004).
• For photographs taken on identical backgrounds and with identical lighting, threshold values can be chosen which allow
  a. differentiation of the leaf from its background hue
  b. differentiation of necrotic/chlorotic tissue from healthy leaf tissue.
• Images are scaled by including objects of known size. Measurements are then provided of selected areas using the “analyse particles” function in ImageJ (Fig. 3a and b).
• Similarly, pycnidial counts can be obtained by thresholding images appropriately and using particle analysis with a low size and high circularity requirement (Fig. 3c).

Note: The image analysis process is amenable to automated batch processing (Stewart and McDonald, 2014). However, this is only viable when precise control of background, lighting and photographic factors (such as focal distance, exposure time) is possible. Indeed, differences in infection severity can alter background ‘healthy leaf’ colouration and different fungal strains can give rise to different levels of melanisation within a lesion, so affecting lesion appearance. Thus, extreme caution should be applied to automated comparisons between datasets.
